# A Rare Manifestation of Abdominal Pain in Systemic Lupus Erythematosus: A Case Report on Lupus Enterocolitis

**DOI:** 10.7759/cureus.82750

**Published:** 2025-04-21

**Authors:** William Vaughan-Williams, Akhil Jerry, Michael Youssef, Syazeddy Samani

**Affiliations:** 1 Internal Medicine, University Hospitals Birmingham National Health Service (NHS) Foundation Trust-Queen Elizabeth Hospital, Birmingham, GBR; 2 Radiology, University Hospitals Birmingham National Health Service (NHS) Foundation Trust-Queen Elizabeth Hospital, Birmingham, GBR; 3 Gastroenterology, University Hospitals Birmingham National Health Service (NHS) Foundation Trust-Queen Elizabeth Hospital, Birmingham, GBR

**Keywords:** comb sign, enterocolitis, lupus enterocolitis, lupus flare, systemic lupus erythematosus

## Abstract

Systemic lupus erythematosus (SLE) is an autoimmune disease that commonly affects the skin, kidneys, and musculoskeletal system. We present a case of lupus enterocolitis, a rare manifestation of SLE that affects the small and large bowel. The case involves a 17-year-old woman with SLE, presenting with a seven-day history of colicky abdominal pain, nausea, and vomiting. A contrast-enhanced CT abdomen and pelvis were organised 48 hours into her admission, showing multifocal inflammatory changes in the small and large bowel, most prominent in the ascending and transverse colon. She was successfully treated with intravenous steroids and continuation of her immunosuppressive agents. This case illustrates that SLE can affect the bowel, leading to an acute abdominal presentation that is difficult to diagnose. Prompt imaging and early administration of intravenous steroids enable effective management of lupus enterocolitis, reducing the risk of life-threatening complications such as bowel ischaemia and perforation.

## Introduction

Systemic lupus erythematosus (SLE) is an autoimmune disease that affects multiple organs. Its presentation ranges from mild mucocutaneous involvement to severe multisystem disease [1]. Gastrointestinal symptoms are a recognised but often overlooked feature of SLE [2]. While they can occur in up to 5.8% of lupus patients, they are frequently attributed to medication side effects, infections, or other concurrent conditions [3,4]. The most common gastrointestinal manifestations in SLE are non-specific; these symptoms include mouth ulceration, dysphagia, nausea, anorexia, and abdominal pain [3]. Rare gastrointestinal manifestations of SLE often mimic more common conditions and present with non-specific symptoms, such as lupus enterocolitis [5-7].

Lupus enterocolitis is a rare but potentially life-threatening manifestation of SLE that results from immune complex deposition in the vasculature of the bowel, triggering vasculitis [8]. This causes microvascular injury and increased vessel permeability, leading to submucosal oedema and inflammation within the small and large bowel [9]. If left untreated, lupus enterocolitis can progress to complications such as bowel ischaemia and perforation [5,8]. Contrast-enhanced CT of the abdomen and pelvis is the first-line diagnostic imaging with characteristic findings such as bowel wall oedema (“target sign”), mesenteric vessel engorgement (“comb sign”), and increased attenuation of mesenteric fat [3,5]. Despite the rarity of lupus enterocolitis, prompt recognition and intervention are critical to prevent severe complications [6]. This case highlights the importance of considering lupus enterocolitis as a differential diagnosis in SLE patients, as timely imaging and corticosteroid therapy can significantly improve patient outcomes [7].

## Case presentation

A 17-year-old woman presented to the emergency department with a seven-day history of right-sided lower abdominal pain, vomiting, nausea, bloating, and reduced oral intake. She initially experienced seven days of nausea and bilious vomiting, followed by intermittent colicky abdominal pain in the right lower quadrant and abdominal bloating around 12 hours later. She denied any associated fevers, loose stools, constipation, or urinary symptoms. On initial assessment, her vital signs were stable, fluid status examination showed reduced capillary refill and dry mucous membranes, and abdominal examination revealed abdominal distention, likely secondary to ascites, with tenderness in the right iliac fossa and active bowel sounds. Furthermore, on presentation, she had no obvious manifestations of SLE, such as oral ulcers, rashes, or arthralgia.

The patient had a three-year history of SLE. On diagnosis, investigations revealed positive ANA, anti-Ro, anti-RNP, anti-Smith, SCL-70, DsDNA, and C1q antibodies, and renal biopsy demonstrated lupus nephritis class V + VI on the International Society of Nephrology/Renal Pathology Society classification for lupus nephritis. Following induction therapy with cyclophosphamide and rituximab upon diagnosis, she was managed with 5mg once daily prednisolone, 500mg twice daily mycophenolate mofetil (MMF), and four-weekly belimumab intravenous infusions.

Her admission blood results, shown in Table 1, revealed a stage two acute kidney injury (AKI), mild microcytic anaemia, mild thrombocytosis, and raised CRP. An infection screen showed negative cultures from blood, stool, urine and ascitic fluid. Urinalysis demonstrated microscopic haematuria, mild proteinuria and a negative HCG. Additional investigations revealed raised dsDNA and IgG levels, low C3 and C4 counts, and a serum-ascites albumin gradient of 1.3g/L, suggesting portal hypertension.

**Table 1:** Admission and follow-up blood test results ALP: Alkaline phosphatase; ALT: Alanine aminotransferase; CRP: C‐reactive protein, eGFR: Estimated glomerular filtration rate, MCV: Mean corpuscular volume

Parameter	Admission Value (Baseline)	Follow-up Value	Reference range (unit)
Haemoglobin	111	88	115-160 (g/L)
MCV	76.6	80.2	80-100 (fL)
Platelets	499	320	150-450 (10^9^/L)
White cell count	6.57	4.39	4-11 (10^9^/L)
Albumin	30	32	45 - 84 (umol/l)
Bilirubin	3	4	0 - 21 (umol/l)
ALP	69	85	30-130(iu/l)
ALT	<6	12	0-33 (iu/l)
CRP	26	6	0-5 (mg/l)
Neutrophils	5.6	3.4	1.8-7.7 (10^9^/L)
Lymphocytes	0.82	0.84	1-4.8 (10^9^/L)
Sodium	133	141	133 - 146 (mmol/l)
Potassium	5.3	4.2	3.5 - 5.3 (mmol/l)
Urea	10.4	3.9	2.5 - 7.8 (mmol/l)
Serum creatinine	116 (47)	38	45 - 84 (umol/l)
Adjusted calcium	2.39	2.38	2.2 - 2.6 (mmol/l)
Glucose	4.7	N/a	5.6 to 6.9 (mmol/l)

**Figure 1:**
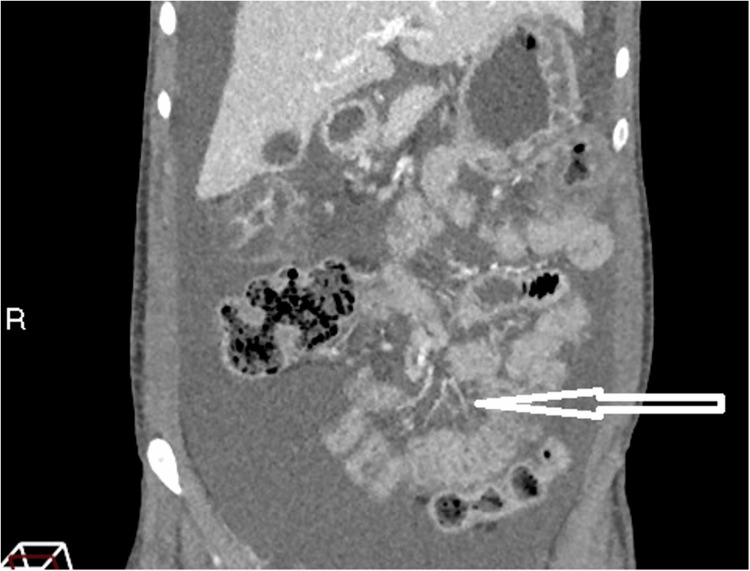
Contrast-enhanced CT of the abdomen and pelvis in the coronal plane showing the “comb sign” The arrow points to the mesenteric vessels. These vessels are abnormally enlarged due to reactive hypervascular filling with blood. This is a response to active inflammation. The appearance of the engorged vessels interspersed with fat (or large volume ascites, as seen in this figure) resembles the appearance of a hair comb. This sign helps differentiate active inflammatory disease from other causes such as metastatic deposits or bowel lymphoma, which appear hypovascular.

**Figure 2:**
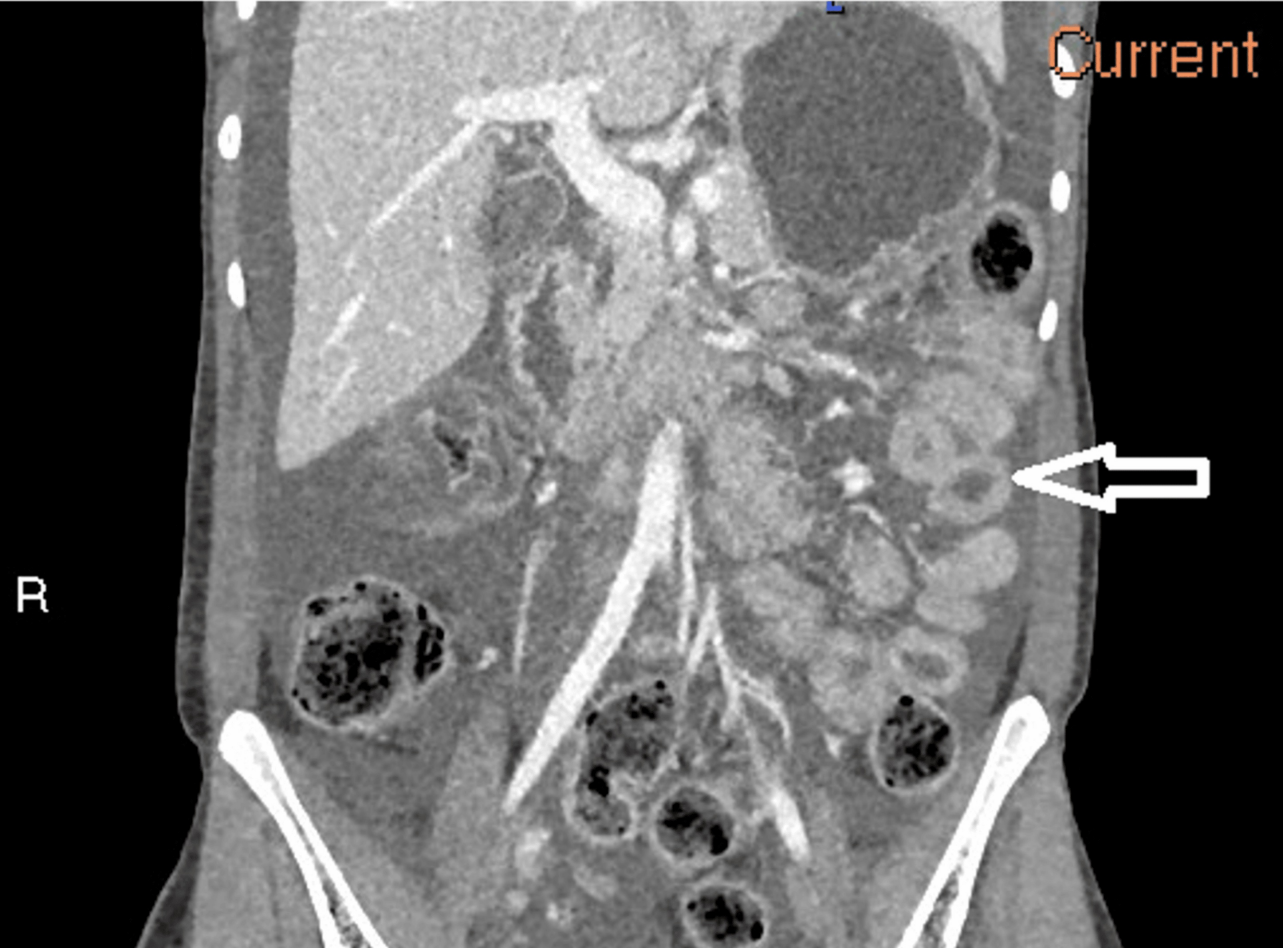
Contrast-enhanced CT of the abdomen and pelvis in the coronal plane demonstrating the “target sign” Coronal cross-sectional images of the abdomen and pelvis show the jejunum (arrow) has circumferential inflammatory thickening of the bowel wall and central hypoattenuation in keeping with oedematous change of the submucosal layer. This manifests radiologically as concentric rings of alternating attenuation, resembling a target, demonstrating the “target sign” (or “double halo sign”).

**Figure 3:**
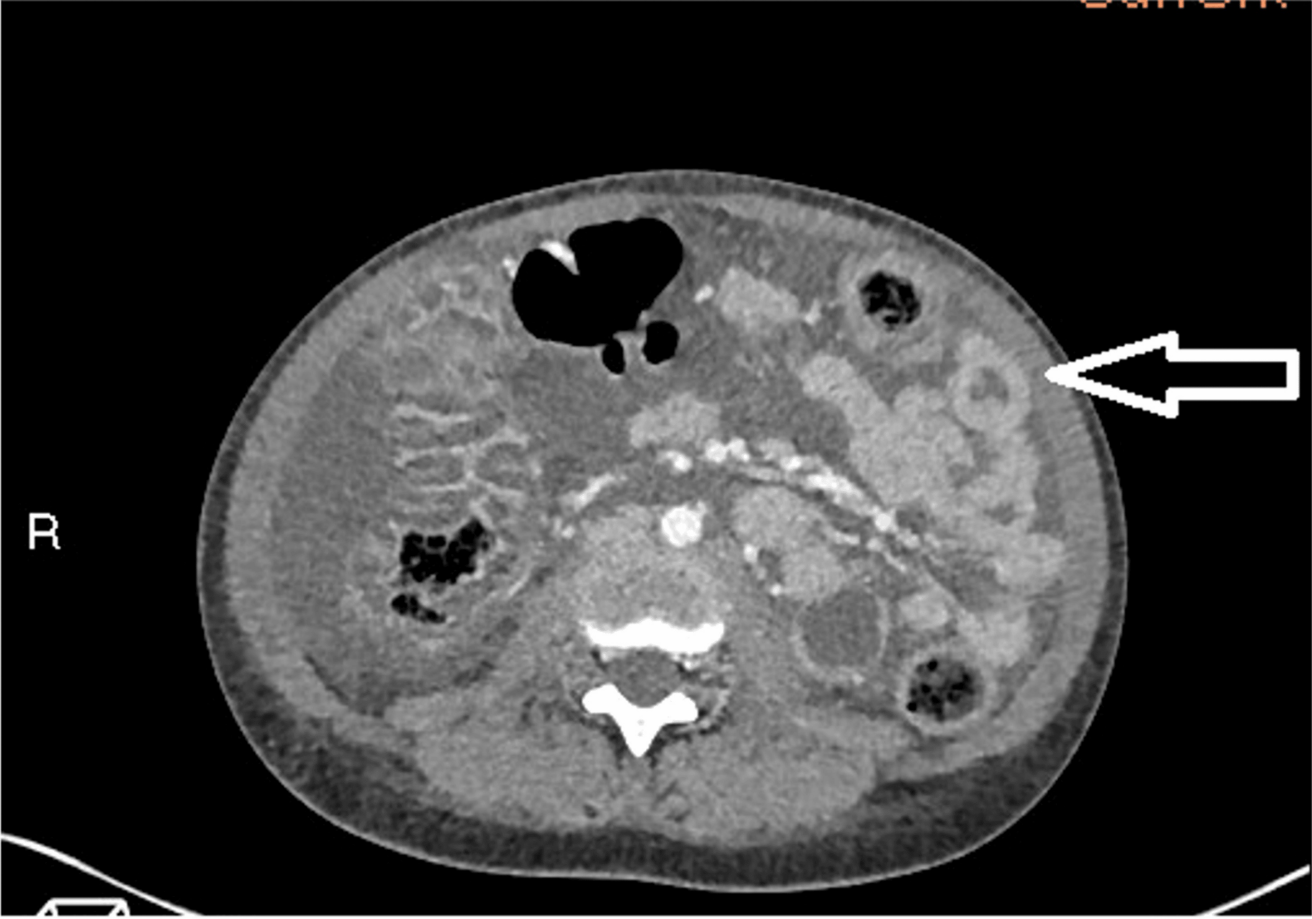
Contrast-enhanced CT of the abdomen and pelvis in the axial plane demonstrating the “target sign” Axial cross-sectional CT image of the abdomen and pelvis showing inflammation within the bowel loops. The jejunum (arrow) shows the typical “target sign”, as described above. Lupus has a predilection for jejunal inflammation. The target sign may also be observed in other active inflammatory conditions such as inflammatory bowel disease, ischaemic bowel and infectious colitis. In lupus, a lack of other typical IBD or neoplastic findings on imaging, plus serological evidence of SLE activity, can guide the correct diagnosis.

**Figure 4:**
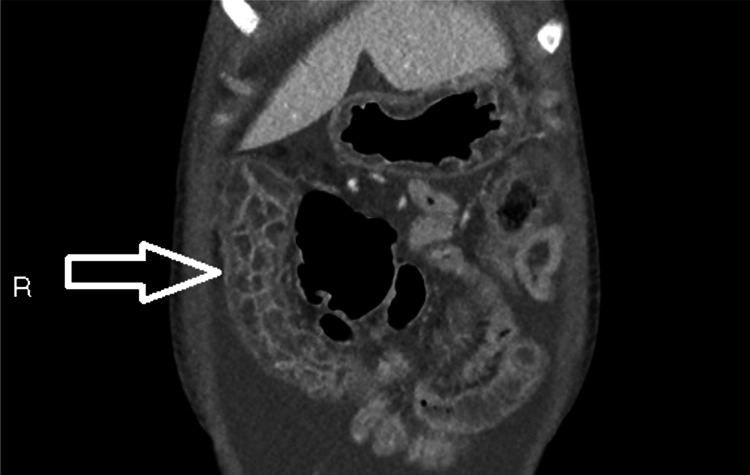
Contrast-enhanced CT of the abdomen and pelvis in the sagittal plane showing transverse colitis The transverse colon (arrow) demonstrates inflammatory colitis; the bowel wall and segmental haustra are thickened and enhanced by hyperaemia. There is significant oedematous change within the mural and submucosal layers, in addition to intra-luminal fluid filling, creating the characteristic appearance of transverse colitis. This figure underscores that lupus enterocolitis can present in various segments of the GI tract.

**Figure 5:**
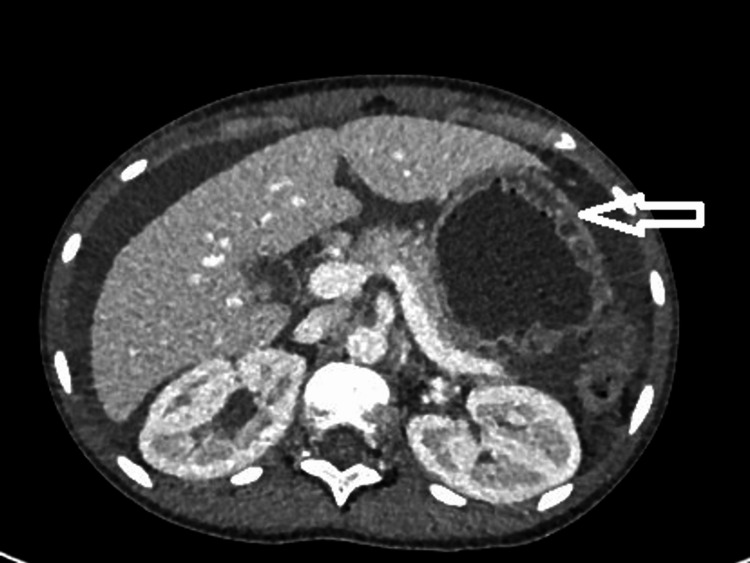
Contrast-enhanced CT of the abdomen and pelvis in the axial plane revealing gastric antral vascular ectasia (“watermelon stomach”) The stomach wall (arrow) has scalloped and prominent antral folds that radiate to the pylorus. The internal vasculature of the stomach is ectatic and enlarged, which follows the fold pattern to congregate towards the antrum. This gives a characteristic “watermelon stomach” appearance. This finding in the context of an inflamed bowel is often associated with autoimmune-related colitis.

The patient was initially managed for gastroenteritis with a pre-renal acute kidney injury (AKI), and by day one of her admission, she reported symptomatic improvement on simple analgesia, with a plan made for discharge. Subsequently, on day two of admission, her abdominal pain and nausea worsened despite supportive treatment. Given she had serological evidence of a lupus flare and partial response to analgesia, we suspected potential lupus-related bowel involvement and therefore proceeded with contrast-enhanced CT imaging. This additionally allowed us to rule out other differential diagnoses, such as appendicitis and inflammatory bowel disease. The images revealed multifocal inflammatory changes in the small and large bowel, suggestive of lupus enterocolitis, plus large volume ascites (Figures 1-5). Inflammation was most prominent in the ascending and transverse colon.

She was initially treated with three days of intravenous pulse methylprednisolone, improving her abdominal pain, nausea, and vomiting. The patient received 500mg of methylprednisolone on day one and 250mg on days two and three. She was additionally given intravenous vitamin B and C complex due to the risk of refeeding syndrome. Gastroenteritis was the initial diagnosis; therefore, MMF was paused due to concerns of immunosuppression but was restarted on the final diagnosis. Following six further days on 10mg once daily oral prednisolone, her symptoms largely subsided, except for mild abdominal distention. She was discharged after nine days, on a reduced course of prednisolone with a lupus clinic follow-up appointment at two weeks. Her follow-up blood results (Table 1) showed an improvement in her inflammatory markers and renal function; however, a drop in her haemoglobin, which slowly increased in repeat blood tests over the next few months. Furthermore, she reported no recurrence of symptoms at follow-up.

## Discussion

We report a case of lupus enterocolitis, a rare gastrointestinal manifestation of SLE [4]. SLE commonly affects the kidneys, skin and musculoskeletal system; nonetheless, it can also affect the bowel [5]. This case exemplifies the need to consider lupus enterocolitis in patients with SLE. The patient was initially diagnosed with a pre-renal AKI secondary to viral gastroenteritis; however, her laboratory investigations and worsening symptom severity warranted additional investigation. Further evaluation prevented the patient from being discharged prematurely, potentially leading to life-threatening complications such as bowel ischaemia or perforation [5]. Complications secondary to untreated lupus enteritis carry a 2.7% mortality rate [10].

Literature suggests that diagnosis of lupus enterocolitis can be challenging given there are no agreed diagnostic criteria [11]. There has been a suggestion that endoscopy and biopsy are of limited value, as usually only superficial tissues are obtained [12]. A literature review found endoscopy had limited sensitivity in detecting lupus enteritis, with more than 50% of cases demonstrating unremarkable macroscopic findings [10].

Imaging is pivotal in the management of lupus enterocolitis. Contrast-enhanced CT of the abdomen and pelvis is the first-line diagnostic imaging, which illustrates three typical findings [3,10,13]. These include increased attenuation of mesenteric fat, bowel wall oedema >3mm (“target sign”), and enlargement of mesenteric vessels (“comb sign”) [9,13,14]. The CT images in this case revealed two of the three criteria, including bowel wall oedema and engorgement of the vessels, with the absence of attenuation of the mesenteric fat. The “Target sign” and “Comb sign” have been reported in up to 71% of cases, and their coexistence together is particularly specific for lupus enterocolitis [14]. However, the images additionally showed evidence of ascites and gastric antral vascular ectasia (GAVE), also known as “watermelon stomach”. Literature suggests both features are associated with lupus-related gastrointestinal involvement, although less characteristic [10,14-16]. The pathogenesis of GAVE in lupus enterocolitis is currently unclear; however, research demonstrates a potential association between autoantibodies cross-reacting with gastric vessels [16]. Bowel disease in SLE carries a high morbidity and mortality rate if diagnosis is delayed, highlighting the importance of prompt imaging [10,14].

Intravenous steroids are the mainstay treatment in this condition and are well tolerated [11,14]. Similarly, as in our case, previous reports document the use of 0.5-1g/day of intravenous methylprednisolone over three consecutive days to induce remission, overall providing successful outcomes [17]. Second-line treatment involves a combination of high-dose steroids and additional immunosuppressants for recurrent or relapsing disease [11,14,18]. There are currently no prospective studies that have investigated the effectiveness of additional immunosuppressive agents in severe lupus enterocolitis. This has caused a lack of consistency in second-line agents administered in previous cases, with a range of agents used, such as MMF, cyclophosphamide, and Infliximab [14,17]. Smith et al. suggest relapse can occur even in patients who initially respond well to steroids [9]. A bowel wall thickness exceeding 9mm is considered a risk factor for relapse and may warrant an extended course of steroid treatment or additional therapy [9]. In this case, the patient’s regular immunosuppressants were paused; however, symptom improvement only occurred once MMF was restarted with the initiation of intravenous steroids. Despite the patient’s symptom improvement at follow-up, she developed worsening anaemia. Case reports have shown anaemia is common in lupus enteritis; however, research has not explored the duration of this derangement post-treatment [3,19].

## Conclusions

There is currently limited evidence of SLE affecting the small and large bowel simultaneously, causing enterocolitis. This case emphasises that clinicians should maintain a high index of suspicion for lupus enterocolitis in SLE patients presenting with gastrointestinal tract symptoms to avoid misdiagnosis and life-threatening complications. Our case demonstrates a lupus patient presenting with acute abdominal symptoms who was initially misdiagnosed as having viral gastroenteritis. Forty-eight hours into her admission, a contrast-enhanced CT of the abdomen and pelvis was organised due to worsening symptom acuity. This is the first-line imaging choice for bowel involvement in SLE. Her images displayed two out of three characteristic findings, instigating the correct diagnosis of lupus enterocolitis. Without prompt imaging, early intravenous steroids, and additional immunosuppression, this patient may have developed complications, such as bowel ischaemia or perforation. There are no agreed diagnostic criteria for lupus enterocolitis. We appreciate the retrospective nature of case reports can be subjective, leading to bias; therefore, we recommend the need for further prospective studies on lupus enterocolitis. Future research should explore developing diagnostic tools and guidelines for lupus-related gastrointestinal involvement to increase awareness and expedite imaging. Thereby, reducing the risk of misdiagnosis and diagnostic delay.

## References

[1] Justiz Vaillant AA, Goyal A, Bansal P (2023). Systemic Lupus Erythematosus. PubMed.

[2] Ronen JA, Mekala A, Wiechmann C, Mungara S (2020). A flare-up of systemic lupus erythematosus with unusual enteric predominance. Cureus.

[3] Potera J, Palomera Tejeda E, Arora S, Manadan AM (2021). Lupus enteritis: an uncommon presentation of lupus flare. Cureus.

[4] Koo BS, Hong S, Kim YJ (2015). Lupus enteritis: clinical characteristics and predictive factors for recurrence. Lupus.

[5] Brewer BN, Kamen DL (2018). Gastrointestinal and hepatic disease in systemic lupus erythematosus. Rheum Dis Clin North Am.

[6] Luís M, Brites AL, Duarte AC (2019). How to diagnose lupus enteritis early? Lessons learned from a multicenter case series. Acta Reumatol Port.

[7] Ling S, Vasant D, Godhamgaonkar V (2024). P38 Lupus enteritis as a first presentation of systemic lupus erythematosus in a previously well patient: a very rare occurrence. Rheumatol Adv Pract.

[8] Almutairi R, Alkhudair D, Aldei A (2025). Lupus enteritis as the early manifestation of systemic lupus erythematosus successfully managed with belimumab: a case report. Cureus.

[9] Smith LW, Petri M (2013). Lupus enteritis: an uncommon manifestation of systemic lupus erythematosus. J Clin Rheumatol.

[10] Janssens P, Arnaud L, Galicier L (2013). Lupus enteritis: from clinical findings to therapeutic management. Orphanet J Rare Dis.

[11] Halilu F, Qureshi A, Nash B (2022). Lupus enteritis in the absence of a lupus flare. A case report and review of literature. J Community Hosp Intern Med Perspect.

[12] Gonzalez A, Wadhwa V, Salomon F (2019). Lupus enteritis as the only active manifestation of systemic lupus erythematosus: a case report. World J Clin Cases.

[13] Jotwani PM, Patel AV (2022). Radiographic findings of lupus related entero-colitis. Clin Rheumatol.

[14] Muñoz-Urbano M, Sangle S, D'Cruz DP (2024). Lupus enteritis: a narrative review. Rheumatology (Oxford).

[15] Kirby JM, Jhaveri KS, Maizlin ZV (2009). Abdominal manifestations of systemic lupus erythematosus: spectrum of imaging findings. Can Assoc Radiol J.

[16] Kang SH, Kim AY, Do JY (2020). Gastric antral vascular ectasia in a patient with lupus undergoing hemodialysis: a case report. BMC Nephrol.

[17] Williamson L, Hao Y, Basnayake C (2024). Systematic review of treatments for the gastrointestinal manifestations of systemic lupus erythematosus. Semin Arthritis Rheum.

[18] Al Balushi F, Humby F, Mahto A (2012). Mycophenolate mofetil inducing remission of lupus enteritis. Lupus.

[19] Zhu XL, Xu XM, Chen S (2019). Lupus enteritis masquerading as Crohn's disease. BMC Gastroenterol.

